# Laparoscopic sleeve gastrectomy under general anesthesia in severely obese patients: a single-centered retrospective study

**DOI:** 10.7717/peerj.10802

**Published:** 2021-02-03

**Authors:** Yuanyuan Ma, Yu Fan, Di Zhou, Junjun Chen, Shengjin Ge

**Affiliations:** Department of Anesthesia, Zhongshan Hospital, Fudan University, Shanghai, China

**Keywords:** General anesthesia, Laparoscopic sleeve gastrectomy, Multidisciplinary collaboration, Severely obese, Tracheal intubation

## Abstract

**Background:**

This study aims to summarize and analyze the clinical characteristics and outcomes of severely obese patients who underwent laparoscopic sleeve gastrectomy (LSG) under general anesthesia with multidisciplinary collaboration.

**Methods:**

A retrospective analysis was performed for 100 severely obese patients who were hospitalized in Zhongshan Hospital, Fudan University from January 2017 to December 2019, and included preoperative general information, laboratory examinations, anesthesia and outcomes.

**Results:**

A total of 100 patients (46 males, 54 females) were admitted to the department of endocrinology: 100 had hepatic steatosis (100%), 43 had sleep apnea hypopnea syndrome (43%), 25 had hypertension (25%), 11 had type 2 diabetes (11%) and 8 had polycystic ovary syndrome (14% of women). The mean age and BMI were 31.52 ± 10.53 years and 43.31 ± 6.80 kg/m^2^, respectively. Visual laryngoscope intubation was successfully performed with routine intravenous induction in the optimum sniffing position at one time. The surgeries were successfully performed under general anesthesia, without conversion, and the operation time was 140.92 ± 31.23 min. The follow-up data for 41 patients were obtained. The postoperative BMI showed a downward trend. The BMI at 1 month and 3 months after surgery were 38.40 ± 6.77 kg/m^2^and 35.52 ± 7.94 kg/m^2^, respectively.

**Conclusions:**

Multidisciplinary collaboration may contribute to better management and recovery during the perioperative period. Visual laryngoscope intubation with intravenous induction was performed successfully in the optimum sniffing position at one time.

## Introduction

Obesity, defined as a body mass index (BMI)>30 kg/m^2^, is often accompanied with sleep apnea hypopnea syndrome, hypertension, diabetes and other diseases and has become a global public health issue  (Tobias et al., 2014). According to the World Health Organization, in 2016, 39% of men and 39% of women aged >18 years were overweight (BMI ≥ 25 kg/m^2^), and 11% of men and 15% of women were obese (BMI ≥30 kg/m^2^). Thus, nearly 2 billion adults worldwide were overweight, and of these, more than half a billion were obese. Nearly one in two adults will have obesity, and nearly one in four adults is projected to have severe obesity by 2020 in America  (Ward et al., 2019). In China, the number of obese men and women was 16.3% and 12.4%, respectively  (Collaboration, 1975).

With the improvement of living standards and changes in living habits in China, obesity has become an important issue. The latest data show that the number of obese people in China has exceeded 100 million. Laparoscopic sleeve gastrectomy (LSG), which reduces the volume of the stomach and maintains the original anatomical structure of the gastrointestinal tract, has been widely used in weight loss surgery for obese patients to improve glucose metabolism and other metabolic disease comorbidities  (Yumuk et al., 2015). Anesthesiologists face specific challenges for obese patients: difficult venous and airway access and the risk of obesity-related comorbidity  (Pouwels et al., 2019).

Since 2015, our center has performed LSG and gradually formed a multidisciplinary collaboration procedure for the perioperative management of obese patients. This study aims to summarize the clinical characteristics, anesthesia management and outcomes to optimize the perioperative management and accelerate the recovery of these patients.

## Materials and Methods

### Background information

Since 2015, laparoscopic sleeve gastrectomy (LSG) for the treatment of obesity has been performed in Zhongshan Hospital, where a multidisciplinary collaboration procedure was gradually formed. The patients were admitted to the Department of Endocrinology to complete the relevant examinations and adjust their medication for comorbidities (including diabetes, hypertension). Preoperative evaluations were conducted by anesthesiologists and surgeons. Some patients with preoperative consultation were adjusted accordingly to arrange the operation. All patients received endotracheal intubation under general anesthesia that was removed in the operating room and observed for 1 to 2 h in the anesthesia recovery room. Then, the patients were transferred to the surgical intensive care unit (SICU) and returned to the general ward after evaluation by the surgeon and intensive care doctor. Some patients who were transferred to the general ward directly met the exit standards of the conventional anesthesia recovery room.

### Study design and Ethics approval

All procedures performed in studies involving human participants were in accordance with the ethical standards of the institutional research committee. This retrospective, observational trial was approved by the Ethics Committee on August 17, 2020 (Approval No: B2020-190). We have gone back to the participants to obtain retrospective permission to use their data. Informed consent form was obtained from all individual participants of this study. The trial was registered before patient enrollment at https://clinicaltrials.gov (NCT04521543).

### Patient population

A total of 100 severely obese patients who underwent laparoscopic sleeve gastrostomy (LGS) under general anesthesia in Zhongshan Hospital from January 2017 to December 2019 were included in this study. The eligibility criteria included patients aged >18 years., American Society of Anesthesiologists (ASA) status II-III and receiving selective laparoscopic sleeve gastrectomy (LSG) under general anesthesia. The exclusion criteria was age <18, BMI <35 kg/m^2^. All patients were allocated to one of the following three groups: group Class I (BMI range 35 to 39.9 kg/m^2^), group Class II (40 to 50 kg/m^2^), and group Class III (>50 kg/m^2^).

### Anesthesia protocol

**Figure 1:**
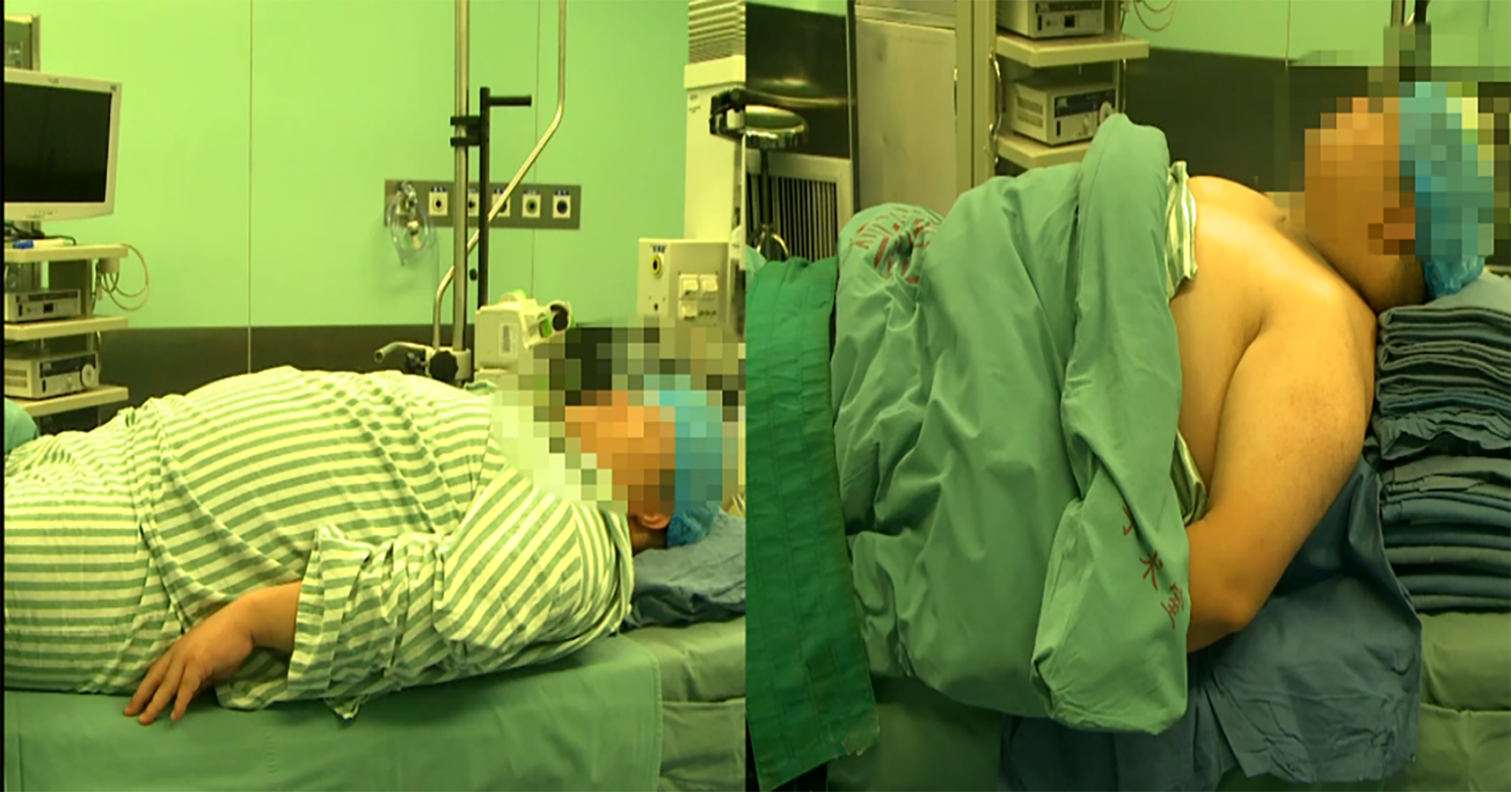
The optimum sniffing position.

All surgeries were performed under general anesthesia by two anesthesiologists. After arriving at the OR, the patients were placed in optimum sniffing position where the shoulders and upper back were elevated to achieve adequate cervical flexion (Fig. 1), which was accomplished using a specialized elevation pillow or folded blankets. Electrocardiogram, invasive blood pressure (radial artery) and pulse oximetry were monitored routinely. Peripheral intravenous cannulation was performed for the administration of fluids and medications, and ultrasound was helpful to locate superficial peripheral veins. General anesthesia was induced with propofol, remifentanil and rocuronium, and the dosage of drugs depended on the patient’s total body weight, lean body weight and ideal body weight. Anesthesia was maintained with intravenous infusion dexmedetomidine and inhalation of 6% to 8% desflurane. Parecoxib was given for multimodal analgesia. The patients used patient-controlled analgesia after surgery.

### Data collection

The data were obtained by querying the electronic medical record system of Zhongshan hospital. The collected data included the following: (1) Patient characteristics, such as age, sex, weight, height, body mass index (BMI); (2) Medical history: chronic diseases, including hepatic steatosis, diabetes, etc.; (3) Laboratory examinations, including fasting blood glucose, 2 h postprandial blood glucose, blood lipids, etc.; (3) Auxiliary examinations, including abdominal ultrasound, lung function, etc.; (4) Anesthesia records; (5) Operation records, hospital stay, complications, etc.; and (6) Follow-up weight loss at 1 month and 3 months after surgery.

### Statistical analyses

Continuous variables are expressed as the mean ± SD and categorical variables as the number or percentages (%). Comparison of numerical variables between groups was made by one-way analysis of variance (one-way ANOVA). The chi-square test or Fisher’s exact test were used for comparisons of categorical data. Significance was expressed by *P*-value, and a value less than 0.5 was considered statistically significant. The data were analyzed using SPSS software version 22.0.

## Results

### Patient characteristics

#### General information

**Figure 2:**
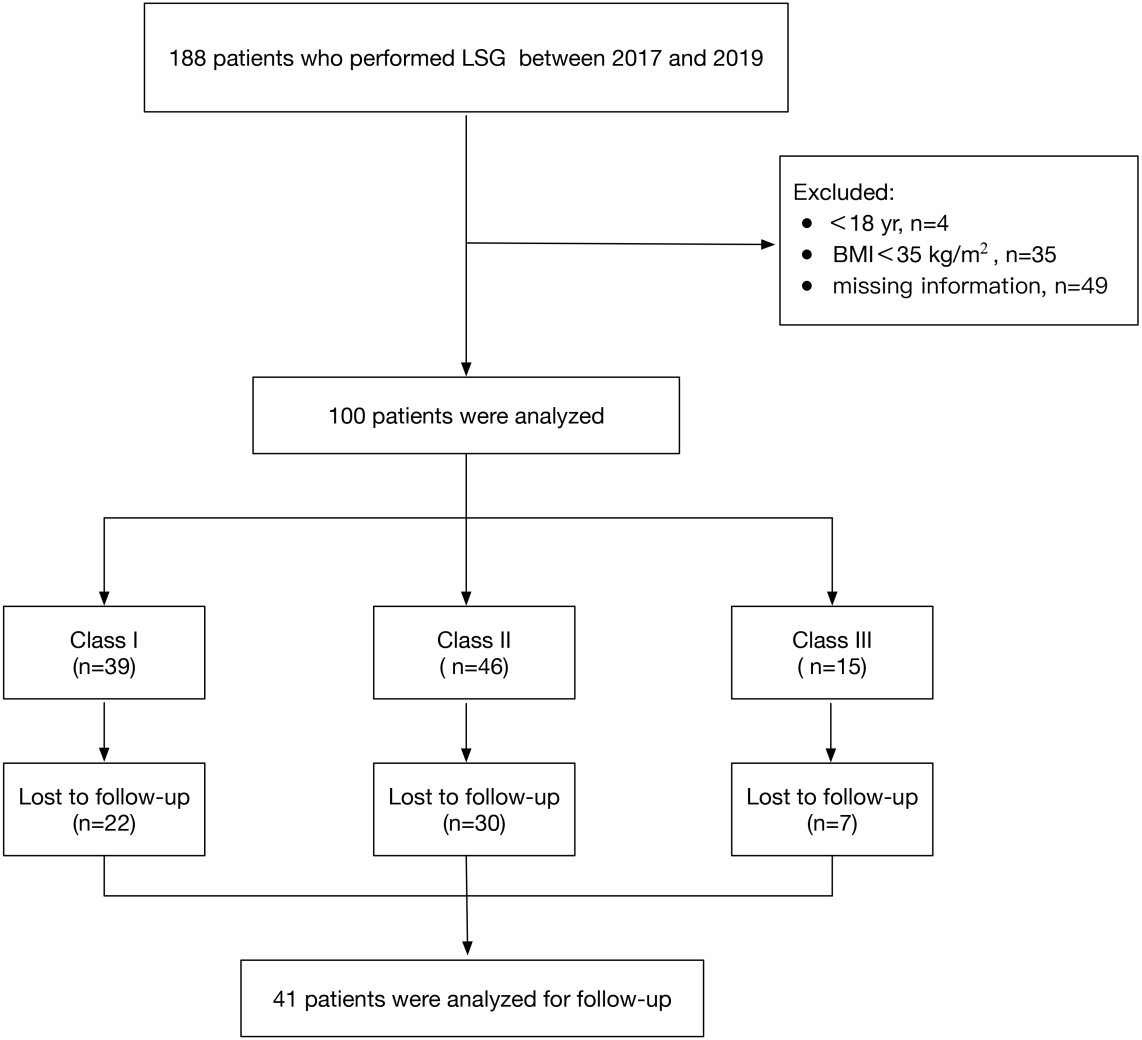
Flowchart of sample.

The participant flow chart is presented in Fig. 2. We excluded patients who were younger than 18 years (4 of 188), had a BMI less than 35 kg/m^2^ (35 of 188) or had missing records (49 of 188). A total of 100 eligible patients were included between 2017 and 2019 (Class I: 39 patients; Class II: 46 patients; Class III: 15 patients) as detailed in Table 1. The patients’ ages ranged from 18 to 64 years, with a mean of 31.52 ± 10.53 years, and their BMI ranged from 35.01 to 63.07 kg/m^2^, with a mean of 43.31 ± 6.80 kg/m^2^. Comorbidities including hepatic steatosis (*n* = 100), sleep apnea hypopnea syndrome (*n* = 43), hypertension (*n* = 25), type2 diabetes (*n* = 11), and polycystic ovarian syndrome (*n* = 8) showed no significant differences between the three groups (*P* > 0.05). A statistically significant difference in sex was found between the three groups: female (66.67%) in Class I, female (54.35%) in Class II and male (80%) in Class III (*P*<0.05). The abdominal ultrasound showed hepatic steatosis in all 100 cases.

**Table 1:** Patient characteristics.

	Class I (*n* = 39)	Class II (*n* = 46)	Class III (*n* = 15)	Total (*n* = 100)	*P*
**General information**					
Age(y)	32.72 ± 10.53	31.30 ± 10.41	29.07 ± 11.12	31.52 ± 10.53	0.517
Male/Female	13/26	21/25	12/3	46/54	0.020
Weight (kg)	103.67 ± 11.89	127.56 ± 14.80^c^	156.39 ± 20.04^a^^b^	122.57 ± 23.15	<0.001
Height (cm)	166.68 ± 8.53	169.31 ± 8.95	167.40 ± 9.59	168.00 ± 8.88	0.385
BMI (kg/m^2^)	37.21 ± 1.50	44.43 ± 2.97^c^	55.73 ± 4.45^a^^b^	43.31 ± 6.80	<0.001
**Comorbidities**					
Hepatic steatosis	39/39	46/46	15/15	100	–
SAHS	9/39	22/46	12/15^a^	43/100	0.101
Hypertension	13/39	8/46	4/15	25/100	0.236
Type2 Diabetes	5/39	5/46	1/15	11/100	0.810
PCOS	4/39	2/46	2/15	8/100	0.431
**Laboratory examinations**					
FBG (mmol/L)	5.28 ± 1.12	5.63 ± 1.93	6.44 ± 3.45	5.62 ± 2.00	0.160
2 h PBG (mmol/L)	9.20 ± 3.35	9.03 ± 3.49	10.81 ± 5.30	9.36 ± 3.77	0.271
HbA1c (%)	5.86 ± 1.12	6.06 ± 1.65	6.70 ± 1.67	6.08 ± 1.48	0.177
TC (mmol/L)	4.66 ± 0.74	4.53 ± 0.82	4.39 ± 0.58	4.56 ± 0.76	0.481
TG (mmol/L)	1.92 ± 0.94	1.73 ± 1.23	1.78 ± 1.38	1.81 ± 1.14	0.736
LDL-ch (mmol/L)	2.83 ± 0.64	2.81 ± 0.67	2.67 ± 0.47	2.79 ± 0.63	0.693
HDL-ch (mmol/L)	1.04 ± 0.29	1.17 ± 0.49	0.95 ± 0.31	1.09 ± 0.40	0.121
FA (mmol/L)	0.51 ± 0.17	0.58 ± 0.16	0.68 ± 0.18^a^	0.57 ± 0.18	0.005

**Notes.**

BMI Body mass index PCOS Polycystic ovarian syndrome SAHS Sleep apnea hypopnea syndrome FBG Fasting blood glucose 2h PBG two hours postprandial blood glucose HbA1c Glycated hemoglobin TC Total cholesterol TG Triglyceride LDL-ch Low density lipoprotein cholesterol HDL-ch High density lipoprotein cholesterol FA Fatty acid

a There was significant difference between Class III and Class I.

b There was significant difference between Class III and Class II.

c There was significant difference between Class I and Class II.

#### Laboratory examinations

The average fasting blood glucose, 2 h postprandial blood glucose and glycosylated hemoglobin levels were 5.62 ± 2.00 mmol/L, 9.36 ± 3.77 mmol/L, and 6.08 ± 1.48%, respectively. The mean total cholesterol, triglycerides, low density lipoprotein cholesterol and high-density lipoprotein cholesterol levels were 4.56 ± 0.76 mmol/L, 1.81 ± 1.14 mmol/L, 2.79 ± 0.63 mmol/L and 1.09 ± 0.40 mmol/L, respectively. No statistically significant differences were detected between the three groups (*P* > 0.05, Table 1); however, the fatty acid level in Class III was significantly higher than Class I (*P* < 0.01, Table 1).

#### Perioperative management

A total of 100 patients were admitted to the department of Endocrinology for preoperative examination and preparation. Visual laryngoscope intubation was performed with routine intravenous induction in the optimum sniffing position. Anesthesia was maintained by combined intravenous-inhalation. LSG were completed by the same group of surgeons without conversion and intraoperative complications. The average operation time was 140.92 ±  31.23 min. The mean blood loss, fluid infusion and urine volume was 61.10 ± 29.61 ml, 1512.00 ± 382.25 ml and 178.80 ± 133.87 ml, respectively (Table 2). All patients were successfully intubated at one time without anesthesia-related complications, such as difficult ventilation, difficult intubation, or hoarseness.

**Table 2:** Intraoperative indexes among three groups.

Parameters	Class I (*n* = 39)	Class II (*n* = 46)	Class III (*n* = 15)	Total (*n* = 100)	*P*
Operation time (min)	127.77 ± 21.23	142.85 ± 28.39^c^	169.20 ± 41.62^a^^b^	140.92 ± 31.23	<0.001
Blood loss (ml)	61.03 ± 29.00	61.09 ± 31.92	61.33 ± 25.32	61.10 ± 29.61	0.999
Fluid infusion (ml)	1405.13 ± 338.69	1530.43 ± 381.73	1733.33 ± 409.99^a^	1512.00 ± 382.25	0.015
Urine volume (ml)	183.46 ± 140.52	178.80 ± 142.40	166.67 ± 87.97	178.80 ± 133.87	0.920

**Notes.**

a There was significant difference between Class III and Class I.

b There was significant difference between Class III and Class II.

c There was significant difference between Class I and Class II.

#### Postoperative indexes

A total of 99 patients were transferred to the surgery intensive care unit (SICU) after observation in the anesthesia recovery room. The average APACHE-II score of SICU admission was 4.64 ±  3.07, and the time of SICU stay was 1.36 ±  0.75 days with no significant difference between the three groups (*P* >  0.05, Table 3). Furthermore, the average postoperative hospital stay, the hospital stay and the hospitalization cost was 7.04 ±  1.95 days, 12.34 ±  2.92 days, and 60694.02 ±  9734.58 yuan, with no significant difference between the groups (*P* > 0.05, Table 3). Only 1 patient returned to the endocrinology ward directly from the postanesthesia care unit and had surgery-related complications (anastomotic leakage).

**Table 3:** Postoperative indexes among three groups.

Parameters	Class I (n=38)	Class II (*n* = 46)	Class III (*n* = 15)	Total (*n* = 99)	*P*
APACHE-II score	4.08 ± 2.84	4.78 ± 3.07	5.60 ± 3.52	4.64 ± 3.07	0.244
SICU stay (day)	1.24 ± 0.63	1.39 ± 0.77	1.60 ± 0.91	1.36 ± 0.75	0.268
Postoperative hospital stay (day)	6.81 ± 2.29	7.14 ± 1.66	7.32 ± 1.92	7.04 ± 1.95	0.622
Hospital stay (day)	11.97 ± 2.90	12.49 ± 2.98	12.86 ± 2.90	12.34 ± 2.92	0.562
Hospital costs (Yuan)	58576.65 ± 9183.57	61186.76 ± 10213.74	64546.98 ± 8732.30	60694.02 ± 9734.58	0.118

**Notes.**

APACHE-II Acute Physiology and Chronic Health Evaluation SICU Surgery intensive care unit

#### Follow-up

One hundred patients meeting the criteria were reviewed; 22 patients in Class I, 30 patients in Class II, and 7 patients in Class III were lost to follow-up. Finally, 41 patients were analyzed for follow-up. The postoperative BMI showed a downward trend, and the difference was statistically significant at 1 month and 3 months (*P* < 0.01). The BMI was 38.40 ± 6.77 kg/m^2^ and 35.52 ± 7.94 kg/m^2^ at 1 month and 3 months after surgery, respectively, without underweight (Table 4).

**Table 4:** BMI in 41 cases severe obesity patients before and after LSG.

	BS	1MAS	3MAS	*P*
Total	43.73 ± 7.14	38.40 ± 6.77^c^	35.52 ± 7.94^a^	<0.001
Class I	37.65 ± 1.39	32.96 ± 2.73^c^	31.83 ± 8.48^a^	0.005
Class II	44.27 ± 2.64	38.94 ± 2.68^c^	35.03 ± 3.87^a^^b^	<0.001
Class III	55.59 ± 4.53	48.85 ± 5.71^c^	44.33 ± 6.41^a^	0.002

**Notes.**

BS Before surgery 1MAS one mouth after surgery 3MAS three mouth after surgery

a There was significant difference between 3MAS and BS.

b There was significant difference between 3MAS and 1MAS.

c There was significant difference between 1MAS and BS.

A total of 11 patients have type 2 diabetes, which controlled the glucose by diet (*n* = 2) or drugs (*n* = 9). However, only 8 patients follow-up data were obtained. The average fasting blood glucose, 2 h postprandial blood glucose (before surgery vs three mouth after surgery) were (8.22 ±  3.06 vs 4.72 ±  0.64)mmol/L and (13.62 ±  4.55 vs 10.08 ±  3.35)mmol/L (*P* < 0.01).

## Discussion

Obesity has become a global health problem, and China has the largest number of obese patients in the world, with approximately 46% of adults and 15% children being obese or overweight  (Wang et al., 2019). Obesity is often accompanied by multiple diseases, including hyperlipidemia, sleep apnea hypopnea syndrome, hypertension, insulin resistance, type 2 diabetes, etc  (Hruby et al., 2016; Arcidiacono et al., 2020). In this study, patients had normal fasting blood glucose levels (5.62 ±  2.00 mmol/L), but they had impaired glucose tolerance with a mean two hour postprandial blood glucose level of 9.36 ±  3.77 mmol/L. The data showed a disorder in blood lipid levels including higher triglycerides (1.81 ±  1.14 mmol/ L) and lower high-density lipoprotein cholesterol (1.09 ±  0.40 mmol/L).

The appropriate initial nonsurgical treatment of obesity is a therapeutic lifestyle change that includes dietary modification, weight loss, physical activity, and treatment of comorbidities such as diabetes, dyslipidemia, and hypertension  (Ruban et al., 2019; Tobias et al., 2015). Surgery has been widely used in obesity to reduce and limit the patient’s food intake capacity, and includes laparoscopic sleeve gastrectomy (LSG), laparoscopic Roux-en-Y gastric bypass (LRYGB), and biliopancreatic diversion with duodenal switch (BPD/DS)  (Lee et al., 2019; Kendrick & Dakin, 2006). Bariatric surgery has increased up to more than 10,000 cases in China. LSG is main method performed in our center. In this study, 100 patients all successfully underwent LSG by the same group of surgeons without conversion, and the weight loss was obvious. Only one patient who has returned directly to the endocrinology from the post anesthesia care is also the one who presented the most feared complication within the gastrectomy sleeve (anastomotic leakage) that resulted in a longer hospital stay. It is difficult to see a link causing direct effect, but SICU observation is perhaps better for patients after LSG.

Obese patients present a special challenge for anesthesiologists regarding airway management, positioning, monitoring, choice of anesthetic technique and anesthetic drugs, pain control, and fluid management  (Pouwels et al., 2019). The preoperative assessment for anesthesia should include consideration of hypertension, diabetes, heart failure, and obesity hypoventilation syndrome. Obesity is commonly perceived to be a risk factor for a difficult airway  (Langeron et al., 2014). An obese patient’s short, thick neck, large tongue, and significant redundant pharyngeal soft tissue usually increases the risk of a difficult airway. One study found an association between oropharyngeal Mallampati classification and BMI as predictors of difficult laryngoscopy  (Voyagis et al., 1998). A meta-analysis of 35 studies showed that the incidence of difficult intubation in obese patients was three times higher compared with a nonobese population  (Shiga et al., 2005). Previous studies in obese patients showed that BMI was not associated with intubation difficulties, and these patients did not have a higher incidence of difficult intubation compared to non-morbidly obese patients with a higher incidence of difficult mask ventilation  (Moon et al., 2019; Wang, Sun & Huang, 2018). The position of an obese individual is important for laryngoscopy and endotracheal intubation  (Troop, 2016). The horizontal alignment of the external auditory meatus with the sternal notch is useful to confirm  (Greenland, Edwards & Hutton, 2010). In our center, the patients were placed in an optimum sniffing position (elevating the upper body and head to align the ear with sternum horizontally) to improve the laryngoscopic view. All patients were successfully intubated endotracheally after one attempt using a visual laryngoscope without difficult mask ventilation or difficult intubation.

Multidisciplinary collaboration (MDC) has been widely applied in the diagnosis and management of several diseases (cancer, pancreatitis) to optimize patient care (Watanabe et al., 2020; Haj-Mirzaian et al., 2020). In 2015, our center created a multidisciplinary team that consisted of several specialists from endocrinology, general surgery, anesthesiology and surgical intensive care unit to establish a coordinated strategy to facilitate the access of patients. Obese patients were admitted to the department of endocrinology and the team would communicate for optimizing treatment of comorbidities such as diabetes and hypertension. The anesthesiologist formulated a suitable individual anesthesia management plan to facilitate the surgeons’ operation and patients’ enhanced recovery. Moreover, our study showed that multidisciplinary collaboration brought benefit to patients.

There are still some limitations in this study. First, this single-center study does not represent the national situation. Second, we only obtained partial follow-up data within a short time frame. Moreover, there are only 100 patients at the beginning and a follow-up limited to 3 months for only 41 patients because of the limited medical records. It might be due to the special medical characteristics of our country which has many provinces and cities with a large population making retrospective research difficult. Our center (Zhongshan Hospital, Fudan University) is a major academic center in Shanghai that many patients were from other cities for treatment. The patients from other provinces and cities often follow-up at the local hospital instead of returning to Shanghai. This is the main reason why some data is not available. Although the study included follow-up data for glucose in some patients with diabetes, data on the outcome of complications such as hypertension and dyslipidemia is still insufficient. Therefore, a multicenter study with a larger sample and longer follow-up is still needed in future.

In conclusion, severely obese patients often have some comorbidities, such as hepatic steatosis, sleep apnea hypopnea syndrome, hypertension, and diabetes. The perioperative multidisciplinary collaboration (general surgery, endocrinology, anesthesiology and SICU) in our center contribute to patient management and recovery. Visual laryngoscope tracheal intubation induced intravenously in an optimum sniffing position could be safely used for severely obese patients, and LSG is safe and effective.

##  Supplemental Information

10.7717/peerj.10802/supp-1 Supplemental Information 1 Raw data

## Additional Information and Declarations

### Data Availability

The following information was supplied regarding data availability: Raw measurements are available as a Supplemental File.
